# Production and Characterization of a New Bacterial Cellulose/Poly(Vinyl Alcohol) Nanocomposite

**DOI:** 10.3390/ma6051956

**Published:** 2013-05-10

**Authors:** Alexandre F. Leitão, João Pedro Silva, Fernando Dourado, Miguel Gama

**Affiliations:** Institute of Biotechnology and Bioengineering, Centre of Biological Engineering, University of Minho, Campus de Gualtar, 4710-057 Braga, Portugal; E-Mails: afleitao@deb.uminho.pt (A.F.L.); jpsilva@deb.uminho.pt (J.P.S.); fdourado@deb.uminho.pt (F.D.)

**Keywords:** bacterial cellulose, poly(vinyl alcohol), mechanical strength, diffusivity

## Abstract

Bacterial cellulose (BC) is characterized for its high water holding capacity, high crystallinity, an ultrafine fiber network and high tensile strength. This work demonstrates the production of a new interpenetrated polymer network nanocomposite obtained through the incorporation of poly(vinyl alcohol) (PVA) on the BC matrix and evaluates the effect of oven drying on the morphological, mechanical and mass transfer properties of the composite membranes. Both the addition of PVA and oven drying induce the appearance of larger pores (*circa* 1–3 µm in average diameter) in dried BC/PVA membranes. Both types of treatments also affect the permeability of the composite, as assessed by the diffusion coefficients of polyethylene glycol (PEG) molecules (900, 8,000, 35,000 and 100,000 Da) across the membranes. Finally, the Young’s modulus of dry pristine BC decreases following PVA incorporation, resulting in a change from 3.5 GPa to 1 GPa and a five-fold loss in tensile strength.

## 1. Introduction

Bacterial cellulose (BC) is produced mainly by *Gluconacetobacter* strains as a hydrated membrane or pellicle at the air-medium interface and represents a promising biomaterial that has been the subject of intensive research and development. It is formed by repeated dimers of β-1,4 linked D-glucose units and reveals unique properties, including high water holding capacity, high crystallinity, an ultrafine fiber network, high tensile strength and a relatively simple, cost-efficient production [1,2]. Moreover, its membrane morphology, along with its physicochemical properties, may be easily manipulated by controlling growth conditions (*in situ* modifications) and/or by chemical modifications (*ex situ* modifications) that allow the desired functionality [3]. 

For the past years, BC has gained interest in the field of tissue engineering, being studied by several research groups as a scaffold for cartilage [4,5], wound dressing [6,7], dental implants [8,9], nerve regeneration [10] and vascular grafts [11,12,13]. The incorporation of living cells into the material is highly desirable; however, the limited porosity precludes proper tissue ingrowth. 

Mass transfer limitations may have a major impact on cell growth and differentiation and even compromise the utility of the scaffold or its usage as a drug delivery device [14,15]. Thus, it is important that a material, once implanted, allows the permeation of water, metabolic products and chemical signals in the aqueous physiological environment [16]. Mass transfer experiments previously conducted by Sokolnicki and colleagues [3], who aimed at determining the transport and interaction parameters of selected molecules through a hydrated BC system, indicated the presence of dual transport mechanisms, for solute transport through the continuous water phase and cellulose matrix, with some hindrance of molecular diffusion via fiber obstruction.

In addition to suitable mass transfer properties, the geometry of the material at the nano- and micro-scales has already been shown to affect cell behavior [17]. For example, the geometry of the microenvironment around osteoprogenitor cells has been shown to regulate the progress of osteoinduction in bone grafts and scaffolds [18]. Thus, the control of BC structure and porosity at the nano and microscales assumes great relevance for applications in which BC is used as a cell scaffold and has been a focus of research [17].

Poly(vinyl alcohol) (PVA) is a synthetic hydrophilic biocompatible homopolymer with desirable characteristics for biomedical applications, such as hemocompatibility and non-linear mechanical properties, both in tension and compression and viscoelastic behavior [19,20,21]. Additionally, it can be physically cross-linked by a low temperature thermal cycling process. This physical cross-linking has the advantage of not leaving residual amounts of any chemical cross-linking agent [22]. Moreover, its mechanical properties can be modified by regulating parameters, like the PVA concentration, the number of freeze/thaw cycles, thawing rate, the freezing holding time and freezing temperature [23]. The combination of PVA and BC has been previously proposed by Millon and Wan [23]; however, these authors used PVA as the matrix for their nanocomposite, adding BC fibers homogenates to the PVA solution. The PVA/BC suspension is then cast to a desired shape. It can then be fine-tuned to exhibit varying mechanical properties via the thermal cycle process [23]. 

Herein, we describe the PVA cross-linking on an intact BC membrane originating a double network where the PVA fibers fill the porous BC matrix. The composite obtained is therefore an interpenetrating polymer network. This composite has been reported before [24], but has remained relatively unstudied. The preparation method we adopted, in regards to impregnation of a BC hydrogel with a PVA solution, is similar to that one presented by Gea and colleagues [24]. However, our nanocomposite differs in terms of PVA concentration, impregnation time and the molecular size of the PVA used. In order to better understand the nanocomposite we are using, we have characterized the surface morphology, permeability, mass transfer and mechanical properties of the composite in this work. To our knowledge, this is the first study to present an interrelation between the morphology, mechanical properties and the permeability of a BC/PVA composite.

## 2. Results and Discussion

### 2.1. Morphology and Characterization of BC and BC/PVA Membranes

**Figure 1:**
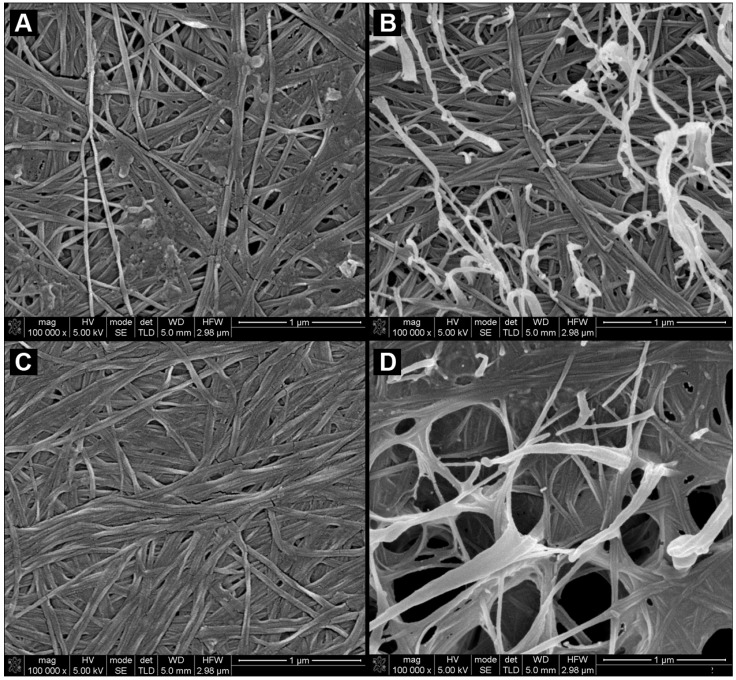
SEM micrographs of (**A**) never-dried bacterial cellulose (BC); (**B**) never-dried BC/poly(vinyl alcohol) (PVA); (**C**) dried BC; and (**D**) BC/PVA at 100,000× magnification.

SEM imaging was used to reveal the morphological features of the BC and BC/PVA membranes. The three-dimensional structure of the BC hydrogel is well documented as a fibrous, porous network of un-oriented nanofibers [5,25,26]. The membranes were freeze-dried so that the three-dimensional morphology would be preserved as in a hydrated state, before and after an initial drying step. The scanning electron micrographs obtained (Figure 1) show the fibrous morphology of the network. All samples presented similar morphological characteristics in terms of the fiber thickness, randomness of fiber distribution and three-dimensional orientation. 

The main and most noticeable differences have to do with the relative dimension and overall number of pores, which is associated with the preparation method and composition of the membrane. The incorporation of PVA (Figure 1b) is clearly noticeable in the composite membranes, although, other than some fibers that project from the membrane surface, the BC and PVA fibers where rather similar, slightly varying in thickness. It was possible to distinguish, to some degree, the PVA from BC fibers in two ways: BC fibers degraded slightly quicker under the electron beam of the SEM than PVA and, also, some from PVA fibers that project and loop from the surface of the BC membrane. These are PVA fibers that formed on top of the BC surface.

**Figure 2:**
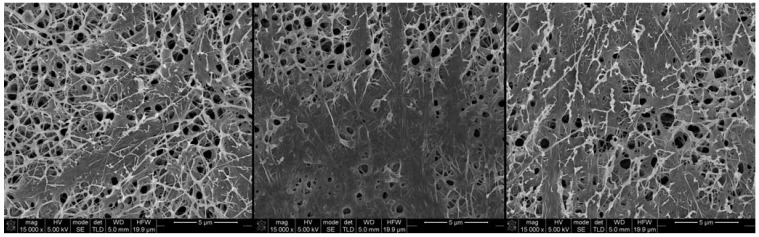
Three SEM micrographs of the same dry BC/PVA membrane at 15,000× magnification with two very distinct regions of densely compacted fibers and regions with large pores.

Previous work had shown that the BC/PVA nanocomposite was made up of interpenetrated fibers [24]. The molecular size and concentration of PVA used does not allow for a PVA hydrogel to form; therefore, rather than a PVA matrix embedding the BC fibers, we have found, as expected, PVA fiber wells integrated into the original BC hydrogel forming an interpenetrated network. The PVA fibers inside the BC membrane randomly bridge individual BC fibers, rising the overall density of the hydrogels. Comparison between the morphology of never-dried (*nd*) (Figure 1a,b) and dried (*d*) (Figure 1c,d) membranes showed clear and marked structural differences. Once BC membranes are dried, the overall thickness of the membranes greatly decreases, resulting in a higher fiber density and, consequently, a much less porous membrane, as can be observed in Figure 1c, when compared to the *nd*BC membrane (Figure 1a). The most remarkable structural difference is observed in the morphology of the *d*BC/PVA membranes (Figure 1d and Figure 2). In this case, there are two very clearly distinct regions comprised of different fiber structures. A large portion of the membrane contained large pores ranging from 500 nm to 2 µm in diameter (in *nd*BC, the largest measurable pores were 120 nm in diameter) that are separated by very densely compacted regions with no discernible pore structures (Figure 2). PVA was largely present in the porous regions, with BC making up much of the denser regions. It should be noted, however, that both BC and PVA fibers could be observed across the entirety of the membrane.

The porous and densely compacted regions of the dried BC/PVA membranes (Figure 2) are due to the presence of PVA. When BC dries, the fibers collapse onto each other and, due to the lack of elasticity, should maintain their overall relative position. When PVA fibers dry or are heated, they contract [27]. As the PVA fibers contract inside the BC network and due to a non-uniform integration of PVA that is associated with the heterogeneity of the BC network, regions with a higher BC fiber density bundle together. The remaining spaces, where the overall number of BC fibers was lower, are bridged by PVA and a few random BC fibers originating in the porous regions.

### 2.2. Mechanical Characterization of Dry BC and Dry BC/PVA Membranes

BC is known for its high tensile strength, with a Young’s modulus’ ranging from 1 MPa to 114 GPa [24,28,29,30,31], varying according to the membrane’s density, dimensions, treatment and preparation methods. Generally, BC is regarded as lacking elastic properties, since hydrogen bonds formed between individual BC strands contribute to the stiffness of the hydrogel. PVA, on the other hand, is known for its elasticity and relatively low tensile strength [24]. As found by Gea and colleagues [24], the impregnation of PVA into the BC network affects the tensile strength of the nanocomposite. This occurs by steric hindrance, where the overall number of hydrogen bonds formed between individual BC fibers during the drying process lowers, due to the presence of PVA [31,32]. 

**Figure 3:**
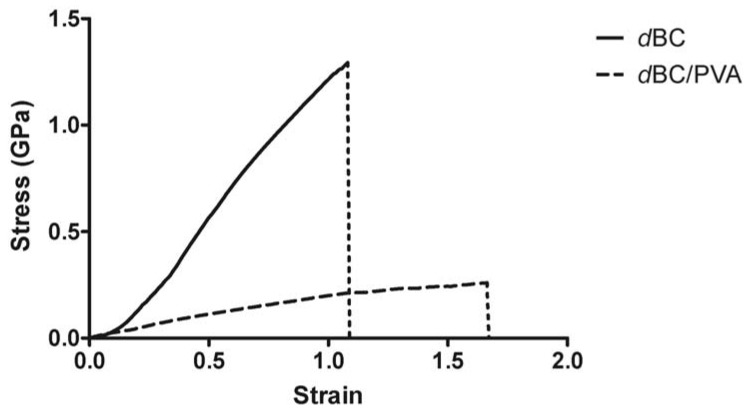
Stress-strain curves of dry strips of BC and BC/PVA. Each strip of 5 × 1 cm (length × width) was cut from a single sheet of material and measured multiple times (*n* = 6). The results here are the averaged curves obtained up to the rupture point.

Our findings are in accordance with this theory. *d*BC resisted better to tensile stress deforming, slightly before abruptly rupturing along the midriff of the strips, with no discernible tearing prior rupture, at a tensile strength of 1.306 ± 0.297 GPa. Whereas, *d*BC/PVA under lower tensile stress started to gradually rip, and so, consequently, tensile strength was nearly five-times lower, 0.271 ± 0.036 GPa. The effect of PVA addition on the Young’s modulus is also noticeable. The Young’s modulus for *d*BC and *d*BC/PVA was, respectively, 3.5 ± 0.5 GPa and 1 ± 0.3 GPa (Figure 3). 

SEM micrographs also present a supporting explanation for the loss in tensile strength. The uneven distribution of PVA across the nanocomposite contributes to the reduction in overall mechanical performance, due to the heterogeneity of the *d*BC/PVA membrane. The *d*BC/PVA membrane presents regions with large pores, which, as mentioned previously, have a lower fiber density, a large portion of which is PVA. The end results of the combination of large pores and high PVA content is that, ultimately, these are mechanically weaker than the surrounding, denser, regions. Therefore the membranes would rupture more easily across these areas. This also explains why the *d*BC/PVA rather than rupturing abruptly, as with *d*BC, tended to rip. The force applied would cause stress failure on the weaker regions, and the uneven impregnation of the BC hydrogel with PVA negatively affected the tensile strength and the Young’s modulus of the nanocomposite. The same did not occur with the *d*BC membranes. In this case, the drying process does not result in higher surface heterogeneity and/or pore distribution. The *d*BC membranes present a uniform and non-oriented distribution of fibers (Figure 1c), which has the end-result of a greater tensile strength and Young’s modulus.

### 2.3. Diffusion Assays

Solute transport has been classified according to two mechanisms: the pore and the partition (or sorption) mechanisms [33,34]. The former states that solutes diffuse through micro-channels in the membrane; as for the partition mechanism, the solute dissolves or is adsorbed onto the membrane itself and diffuses through or along fibers. As described above, the BC and BC/PVA hydrogels are more porous in nature, forming a series of channels that would allow for solute diffusion across the membrane via the pore mechanism. 

As to test the effect of the structural and morphological changes incurred by the drying process on the mass transfer properties, the diffusion across the never-dried (*nd*) and dry (*d*) states of the materials was tested using PEG probes and the diffusion coefficients determined using the time-retention method. It would be expected that the denser the membrane (as is the case with the dry membranes), the harder it would be for solutes to diffuse across the membrane, since the overall interconnection of pores and channels in the membrane would be much lower [35]. Additionally, molecule size would also effect diffusion across the membranes, given the low diffusion coefficient in the liquid phase and also because molecule collision with the fibers would increase with molecule size.

The higher the value for the diffusion coefficient, the more permeable the membrane is and the easier solutes pass through the membrane matrix. Our results show that in both the case of BC and BC/PVA, solute diffusion across the never-dried membranes is easier than in the dried membranes (Table 1). The *nd*BC membranes presented the highest diffusion coefficient values and, so, were the most permeable of the membranes with *nd*BC/PVA second, followed by *d*BC/PVA and, finally, *d*BC. 

**Table 1:** Diffusion coefficients of four different M_w_ polyethylene glycol (PEG) in BC and BC/PVA samples.

PEG M_w_ (Da)	Diffusion coefficient (cm^2^/sec)			
	*nd*BC	*d*BC	*nd*BC/PVA	*d*BC/PVA
900	7.40 × 10^−5^	1.12 × 10^−7^	4.48 × 10^−5^	1.16 × 10^−5^
8000	2.71 × 10^−5^	1.79 × 10^−8^	1.28 × 10^−5^	1.88 × 10^−6^
35,000	2.27 × 10^−5^	1.53 × 10^−8^	1.10 × 10^−5^	1.68 × 10^−6^
100,000	1.95 × 10^−5^	1.41 × 10^−8^	9.82 × 10^−6^	1.50 × 10^−6^

These results can mostly be explained due to the fiber density of the materials. A less dense material will have less fibers in the transport path [3]. *nd*BC was therefore expected to be the most permeable of the membranes. Similarly, the second least dense hydrogel was the *nd*BC/PVA nanocomposite that, despite the formation and entanglement of PVA nanofibers in the BC network (thus, adding to the overall number of fibers), still allows for a porous structure similar to that of *nd*BC. The two denser materials would be the dry membranes, due to the aforementioned collapse of fibers onto each other, thus blocking and reducing in number the pathways available for solute diffusion. However, in the case of the theoretically densest sample, *d*BC/PVA, the diffusion coefficient values present as the third least permeable material. As revealed by the SEM imaging (Figure 2), the two very distinct regions observed are the reason for this occurrence. While, in all the other cases, the pores, regardless of their number, distribution and size, are distributed randomly across the entirety of the 3D-structure of the membrane. Contrarily, on the *d*BC/PVA, there are very compact regions and others with very large pores. Solute diffusion is facilitated across these extremely porous regions, but hindered in the densely compacted regions. Apparently, the balance between densely compacted and porous regions must slightly tend towards the latter, due to the delay in retention time and, consequently, reduction of the diffusion coefficient. 

Our results are similar to the findings of Sokolnicki [3], which tested the diffusion of dextran molecules and showed that molecular size has little effect on diffusion. Higher molecule size dictates collisions with the hydrogel fibers, temporary immobilization and/or possible rerouting around narrowed passages, according to each. The larger the molecule, the likelier it is for these interactions to occur, resulting in a delay in diffusion times. The overall number of pores and pathways across the materials, due to overall fiber number and density, seems to be the main factor responsible for a slower diffusion. This means that diffusion in these membranes is mainly governed by the pore mechanism. 

## 3. Experimental Section 

### 3.1. Bacterial Cellulose and Bacterial Cellulose/Polyvinyl Alcohol

*Gluconacetobacter xylinus* (ATCC 700178) was grown in a modified Hestrin-Schramm medium, supplemented with 2% Corn Steep Liquor and 0.6% ethanol, at pH 5.0. Inoculated Erlenmeyer flasks were then placed in an incubator, and the culture was allowed to grow for 7 days, at 30 °C, under static conditions. The resulting BC membranes were washed thoroughly with distilled water and further purified with 4% NaOH at 60 °C for 90 min, after which they were again washed thoroughly with distilled water until the pH of the membranes was the same as that of distilled water. The membranes where then cut thinly into 2–3 mm thick membranes.

In order to produce the BC/PVA nanocomposite, the purified BC membranes were immersed in a 10% PVA solution (Mw = 30–50,000 g/mol) for 24 h at 80 °C. The membranes were then frozen at −20 °C for 24 h, after which they were thawed at room temperature in distilled water and washed to remove any excess PVA from the membranes.

Dry BC and BC/PVA samples were obtained by thoroughly drying the samples in an incubator oven at 50 °C for a minimum of 12 h. The membranes obtained through this drying process suffer a significant loss in thickness as compared to the never dried membranes, while only slight variation in thickness was registered in the freeze-dried samples.

### 3.2. SEM Imaging

BC and BC/PVA samples were prepared in 2 ways: “dried” (*d*) at 50 °C overnight, so as to remove all residual water, and then rehydrated before freeze-drying; or “never-dried” (*nd*), which were freeze-dried directly after production and then sputter-coated with a gold/palladium mixture. Each sample, whether dried first or maintained wet until freeze-drying, was initially 3 mm thick. The sample’s morphology was then observed with a Nova NanoSEM 200 Scanning Electron Microscope operating at 5 kV (FEI Europe, Eindhoven, The Netherlands).

### 3.3. Stress-Strain Analysis

Dry BC and BC/PVA strips, with 5 × 1 cm, were cut from a single sheet of each material and then analyzed using on a Shimadzu AG-X 50 kN at a traction speed of 5 mm/min and a load cell of 1 kN. Each sheet was initially 1.5 cm thick and then dried at 50 °C overnight, so as to remove all the water. BC strips were on average 0.065 ± 0.005 mm thick, whereas BC/PVA strips were on average 0.480 ± 0.050 mm thick. Six replicates per sample material were tested in order to obtain a significant set of data. Special care was taken in order to attempt to prevent sample slipping from the hydraulic grips by using both fine grain sandpaper and construction paper. 

### 3.4. Diffusion Assays 

Diffusion assays were carried out using custom-built diffusion cells. Each cell consisted of a donor chamber and a receptor chamber separated by a membrane of either never-dried (nd) or dry (d) BC or BC/PVA with 9.61 cm^2^. Four different molecular mass polyethylene glycol (PEG) solutions were used in these assays: 900, 8000, 35,000 and 100,000 Da at a concentration of 50 mg/mL.

Each donor chamber was filled with 3.5 mL of PEG solution and the receptor chamber with 3.5 mL of distilled water. 100 µL samples were taken at specific intervals to determine the retention time for each of the solutions. The refraction index of the samples was measured on a Knauer RI Detector K-2300 (Knauer) connected to Pharmacia LKB LCC-500 Plus FPLC (GE Healthcare Life Sciences) in order to obtain the diffusion profiles for each molecular weight PEG through n*d*BC, *d*BC, *nd*BC/PVA and *d*BC/PVA.

Membrane thickness (L) was determined with a standard outside micrometer at three different locations on each membrane and then averaged. The diffusion coefficients (D) were calculated by testing the retention time, estimated from the slope of the linear regressions in the diffusion curves (θ), before running into the balance state, according to the following equation:

D = L^2^/6θ (1)

### 3.5. Reagents

All reagents used were acquired from Sigma-Aldrich Quimica, S.L. (Sintra, Portugal).

## 4. Conclusions 

Previous researchers had shown that a BC hydrogel impregnated with PVA had some potential in biomedicine, namely in cardiovascular applications. The work we present here shows the mechanical, permeability and morphology changes incurred by both incorporation of PVA, in a different concentration and molecular size than previously proposed, and the effect of drying the materials. 

Both *nd*BC and *nd*BC/PVA are similar in terms of morphology of the hydrogels and fiber distribution, with an increase in overall fiber number in BC/PVA. When dried, the morphology of both materials change, BC fibers collapse, forming a denser membrane, which leads to a decrease in the permeability. Dry BC/PVA membranes suffer dramatic changes in terms of morphology, creating two distinct regions: densely compacted regions leaving other regions mostly consisting of PVA fibers with large pores. The dual nature of the dried BC/PVA membrane affects both the permeability and mechanical behavior of the material. The tensile strength of BC decreases, due to the presence of PVA impeding the interactions of BC fibers and creating large porous regions that are easier to rupture. Potentially, these large pores could serve in aiding cell migration into the material and nutrient diffusion across the membrane. Therefore, we here show that by promoting physical changes and/or altering the composition of a BC hydrogel, it is possible to alter its morphology, mechanical characteristics and permeability.
